# Long-term clinical outcomes of gastric mucosa-associated lymphoid tissue lymphoma in real-world experience

**DOI:** 10.1007/s00277-023-05130-8

**Published:** 2023-02-25

**Authors:** Gi-June Min, Donghoon Kang, Han Hee Lee, Seung-Jun Kim, Tong Yoon Kim, Young-Woo Jeon, Joo Hyun O, Byung-Ock Choi, Gyeongsin Park, Seok-Goo Cho

**Affiliations:** 1 Department of Hematology, Catholic University Lymphoma Group, Seoul St. Mary’s Hospital, College of Medicine, The Catholic University of Korea, Banpo-Daero 222, Seocho-Gu, Seoul, Republic of Korea; 2 Department of Gastroenterology, Seoul St. Mary’s Hospital, College of Medicine, The Catholic University of Korea, Banpo-Daero 222, Seocho-Gu, Seoul, Republic of Korea; 3 Department of Gastroenterology, Yeouido St. Mary’s Hospital, College of Medicine, The Catholic University of Korea, 10, 63-Ro, Yeongdeungpo-Gu, Seoul, Republic of Korea; 4 Department of Gastroenterology, H+ Yangji Hospital, Nambusunhwan-Ro, 1636 Gwanakgu, Seoul, Republic of Korea; 5 Department of Hematology, Catholic University Lymphoma Group, Yeouido St. Mary’s Hospital, College of Medicine, The Catholic University of Korea, 10, 63-Ro, Yeongdeungpo-Gu, Seoul, Republic of Korea; 6 Department of Nuclear Medicine, College of Medicine, Catholic University Lymphoma Group, Seoul St. Mary’s Hospital, The Catholic University of Korea, Banpo-Daero 222, Seocho-Gu, Seoul, Republic of Korea; 7 Department of Radiation Oncology, Catholic University Lymphoma Group, Seoul St. Mary’s Hospital, College of Medicine, The Catholic University of Korea, Banpo-Daero 222, Seocho-Gu, Seoul, Republic of Korea; 8 Department of Hospital Pathology, Catholic University Lymphoma Group, Seoul St. Mary’s Hospital, College of Medicine, The Catholic University of Korea, Banpo-Daero 222, Seocho-Gu, Seoul, Republic of Korea

**Keywords:** Gastric MALT lymphoma, *Helicobacter pylori*, Eradication, Radiotherapy, Chemotherapy, Prognosis

## Abstract

This long-term, retrospective, single-center study evaluated real-world clinical outcomes of gastric mucosa-associated lymphoid tissue (MALT) lymphoma using different therapeutic modalities and analyzed factors affecting survival outcomes and long-term prognosis. We enrolled 203 patients with pathologically confirmed low-grade gastric MALT lymphoma and examined their treatment responses. *Helicobacter pylori* eradication was performed in all patients with *H. pylori* infection (HPI) and localized stage gastric MALT lymphoma. All patients underwent pre-treatment and physical evaluations, with complete blood count, biochemistry panel, and staging workup. Among 144 HPI-positive patients with stage I or II1–2 disease who underwent *H. pylori* eradication, 112 (77.8%) achieved complete remission (CR). All HPI-negative patients who received first-line radiotherapy achieved CR (100%), but only 22 of 27 first-line chemotherapy-treated patients achieved CR (81.5%). Lesions in the proximal upper-third or in multiple locations and an invasion depth to the submucosa or deeper were associated with poor response to eradication, and HPI negativity was significantly correlated with poor progression-free survival. HPI eradication treatment should be the first-line treatment for patients with localized stage HPI-positive gastric MALT lymphoma. The “watch-and-wait” strategy should be adopted for delayed responders. We suggest radiotherapy for patients with a localized HPI-negative status or when eradication has failed.

**Supplementary Information:**

The online version contains supplementary material available at 10.1007/s00277-023-05130-8.

## Introduction

The incidence of gastric mucosa-associated lymphoid tissue (MALT) lymphoma is increasing, and it accounts for approximately 40–50% of gastric lymphomas, 30–40% of extranodal lymphomas, and 1–6% of all gastric malignancies [1, 2]. Its pathogenesis is related to *Helicobacter pylori* infection (HPI), and the presence of HPI determines the therapeutic approach for its treatment [3, 4]. The role of HPI in gastric MALT lymphoma tumorigenesis is associated with chronic inflammatory stimulus, and Wotherspoon et al*.* first showed that HPI eradication resulted in lymphoma regression [5–7]. The prognosis is known to be favorable, and eradication of *H. pylori* is recommended as the initial treatment for HPI-positive gastric MALT lymphoma. Both radiation and chemotherapy are suitable alternatives for HPI-negative, relapsed, refractory, or high-grade gastric MALT lymphoma. At the localized stage, HPI eradication is potentially curative and results in gastric MALT lymphoma regression in 60–80% of the cases [1, 8, 9]. However, approximately 10% of the gastric MALT lymphoma patients at the early stages with HPI-negative or t(11;18)(p21;p21)/*API2-MALT1* positive findings showed an inadequate response to eradication, and the best treatment strategy for these patients remains controversial [1, 10, 11]. Considerable treatment options for HPI-negative or eradication-resistance gastric MALT lymphoma include endoscopical or surgical resection, chemoimmunotherapy, or radiation. In this study, we describe real-world clinical outcomes of the management of gastric MALT lymphoma using different therapeutic modalities in patients with various demographic and endoscopic characteristics. We also analyzed the factors that affect survival outcomes and long-term prognosis.

## Methods

### Patient enrolment, diagnosis, and staging workup

This single-center retrospective analysis enrolled 203 patients diagnosed with gastric MALT lymphoma between September 2001 and August 2020 at the Catholic University Lymphoma Group (CULG). Specimens were obtained by esophagogastroduodenoscopy with multiple biopsies taken from the gastric, junctional gastroesophageal regions, and any other sites with perceptibly different morphology. Expert pathologists confirmed the diagnosis of gastric MALT lymphoma by histopathological evaluation of the gastric biopsies according to the morphological as well as immunophenotypic diagnostic criteria of lymphoma as defined by the World Health Organization classification and compatible with Wotherspoon's histological score of 3–4 or 5, confirming B-cell monoclonality by immunoglobulin heavy chain rearrangement analysis [12, 13]. The macroscopic type of lymphoma was determined based on endoscopic findings and classified as superficial erosion or ulcer, hypertrophic fold, ulceroinfiltrative, and ulcerofungating type [4, 14]. Gastric MALT lymphomas were also classified as proximal upper-third (cardia, fundus, upper body), distal two-thirds (midbody, lower body, antrum, and pylorus), or diffuse lesions, according to their location in the stomach [1, 4].

Pre-treatment evaluation, physical examination, complete blood count, as well as a biochemistry panel including liver and renal function, lactate dehydrogenase and beta-2 microglobulin levels, serum immunofixation, and the serologic status of chronic hepatitis B and C were evaluated according to the current guidelines [8]. Staging workup was performed using computed tomography (CT) of the neck, chest, abdomen, and pelvis, combined with bone marrow biopsy and fluorine-18 fluorodeoxyglucose positron emission tomography-CT (18F-FDG PET-CT) to detect distant lymph nodes or organ involvement. All enrolled patients were stratified using the MALT-lymphoma International Prognostic Index (MALT-IPI) with the modified Ann Arbor system by Musshoff to identify patients at risk of poor outcomes [15, 16]. We also utilized the Lugano and Paris TNMB staging system designed for gastric MALT lymphoma, more accurately demonstrating the involvement of the depth of the gastric wall, which may predict the response of the lymphoma to HPI eradication or decide the appropriate treatment modality in the advanced stage [17, 18]. We defined advanced-stage gastric MALT lymphoma as the Lugano stage II2, IIE, and IV, and localized-stage gastric MALT lymphoma as the Lugano stage I and II1. The detailed modified Ann Arbor system by Musshoff and the Lugano and Paris TNMB staging system are presented in Online Resource 1.

The following tests determined the presence of HPI: (1) Warthin–Starry, Giemsa stain, or polymerase chain reaction (PCR) with electrophoresis in the gastric biopsy sample, (2) rapid urease test (Campylobacter-like organism test), or (3) 13C urea breath test combined with positive *H. pylori* IgG serology. Patients with at least one positive test result were defined as HPI-positive. If the presence of active HPI was not demonstrated by histochemistry, it was ruled out on showing negative results in serology and the 13C urea breath test [19]. We also used endoscopic ultrasound (EUS) to evaluate the tumor invasion depth and degree of perigastric lymphadenopathy for a more accurate diagnosis, staging, and prognosis prediction. To detect the t(11;18)(p21;p21)/*API2-MALT1* translocation, we performed fluorescence in situ hybridization studies that may be useful in identifying patients who might respond poorly to HPI eradication [19].

### Treatment strategy

The primary therapeutic modalities were determined using the Lugano and Paris staging system (Online Resource 1) and the HPI status. *H. pylori* eradication was performed in all patients with HPI and localized stage gastric MALT lymphoma. For first-line eradication therapy, a proton pump inhibitor (PPI)-based triple therapy regimen was administered for 2 weeks: PPI (standard dose twice a day), clarithromycin (0.5 g twice a day), and amoxicillin (1 g twice a day). 13C urea breath tests were performed in all patients for 3 months or at least 8 weeks after treatment completion, and at least 2 weeks after PPI withdrawal to confirm HPI eradication. For patients who failed first-line triple therapy, a second-line quadruple-therapy regimen consisting of PPI (standard dose twice a day), tripotassium dicitrato bismuthate (300 mg four times a day), metronidazole (500 mg thrice a day), and tetracycline (500 mg four times a day) was administered for 1–2 weeks.

Patients received radiotherapy, chemotherapy, or chemoradiotherapy if they did not achieve lymphoma regression following first- and second-line HPI eradication therapy, or were at the localized stage without initial HPI, or had advanced-stage gastric MALT lymphoma. For radiotherapy, the clinical target volume included the entire stomach and regional lymph nodes and was prescribed as 30.6 Gy over 17 fractions on the stomach [20]. The internal target volume (ITV) and planning target volume were set using the motion information obtained from the 4-dimensional CT for assessment of breathing motions and defined as an expansion of 5 mm from the ITV considering the set-up error of the patient [20]. Patients with the involvement of ≥ 2 organs were excluded from radiotherapy. The R-CVP was the primary systemic chemotherapy regimen, consisting of rituximab 375 mg/m^2^, cyclophosphamide 750 mg/m^2^, and vincristine 1.4 mg/m^2^ on day 1, and prednisolone 60 mg/m^2^ on days 1–5 every 21 days. Localized stage lesions involving small-sized mucosal layers in patients with initial HPI-negative findings could be selectively treated by endoscopic mucosal resection (EMR) and close observation. In the case of chemoradiotherapy, we only used additional radiotherapy for consolidation purposes after chemotherapy by the physicians’ decision. To investigate the side effects of each treatment modality, we reviewed the medical records following the National Cancer Institute’s Common Terminology Criteria for Adverse Events version 5.0.

### Response assessment

Based on the European Gastro-Intestinal Lymphoma Study grading system, the post-treatment response was classified into four groups [19]. Complete remission (CR) was defined as no macroscopic findings of lymphoma as well as negative histologic findings in at least two subsequent follow-up investigations, and partial remission (PR) was defined as at least a 50% reduction in macroscopic tumor and its corresponding histologic findings. Stable disease (SD) and progressive disease (PD) were defined as unmodified macroscopic and/or histologic findings and worsening macroscopic and/or histologic findings, respectively.

Histological evaluation by repeated gastroendoscopic biopsy is an essential follow-up procedure to exclude the possibility of persistent disease, especially in patients with persistent HPI. In the HPI eradication group, approximately 3 months after the completion of treatment, endoscopic follow-up with multiple biopsies was performed for the histologic and cultural evaluation of *H. pylori* along with the 13C urea breath test. We identified the residual presence or no regression of mucosal lesions based on endoscopic biopsy findings and pathological review at 3 and 6 months. Histological evaluation of post-treatment biopsies was performed by reviewing previous biopsy samples to assess cellular infiltration, lymphoepithelial lesions, and stromal changes according to the Groupe d’Etude des Lymphomes de l’Adult (GELA) grading system [21]. In the GELA category, clinical CR can be subdivided into complete histological response (ChR) and probable minimal residual disease (pMRD). Responding residual disease (rRD) and no change (NC) were considered clinically PR and SD, respectively [21]. Details of the GELA grading system for post-treatment evaluation of gastric MALT lymphoma are presented in Online Resource 2.

**Fig. 1:**
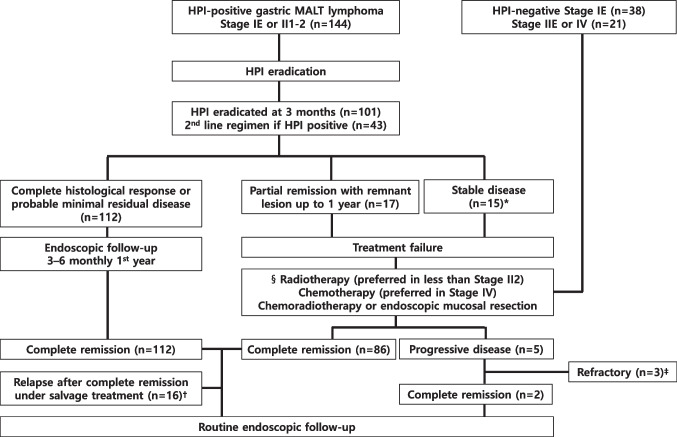
The consort diagram of enrolled gastric MALT lymphoma patients’ treatment algorithms for either localized or advanced stage (*N* = 203). *Antibiotic resistance or no lymphoma response at repeat EGD 3 months after eradication treatment. §Detailed treatment outcomes are presented in Table 2 (first line) and Online Resource 2 (second line). †One patient died during second-line chemotherapy due to septic shock; the other patients achieved second CR. ‡Among three refractory patients, one patient died after autologous HSCT because of septic shock. The other two patients were under the best supportive care only

HPI eradication responders were defined based on post-treatment biopsy histology showing ChR or pMRD in at least two subsequent follow-ups. For delayed responders (rRD), we watched and waited for an additional 6 months without further treatment until 1 year. Patients with NC, eradication failure after second-line eradication, macroscopic SD/PD, or rRD with PR in macroscopic findings up to 1 year were determined as non-responders with treatment failure. They then received radiotherapy, chemotherapy, or chemoradiotherapy as salvage treatments. Regular (3–6 monthly) endoscopic surveillance with imaging workup (neck, chest, and abdomen CT) during the 1st and 2nd years under the discretion of a clinician, 6 monthly during the 3rd to 5th year, and yearly thereafter was performed in patients with CR. The response surveillance protocol was the same in HPI-negative patients, and the treatment algorithms summarizing the management strategies discussed above for either early or advanced gastric MALT lymphoma are presented in Fig. 1.

### Statistical analysis

Overall survival (OS) was measured from the date of treatment initiation to the date of death from any cause or the date of the last follow-up, and progression-free survival (PFS) was measured from the date of treatment initiation to treatment failure, relapse, last follow-up date, or death due to any reason. Cumulative incidence of relapse (CIR) and treatment-related mortality (TRM) were also measured from the treatment initiation date, using cumulative incidence estimation that considered death without evidence of disease recurrence and relapse or death unrelated to treatment as competing risks. The Kaplan–Meier method was used to calculate OS and PFS, and between-group comparisons were performed using the log-rank test. Gray’s test was used to analyze TRM and CIR. We utilized the Cox or the Fine–Gray proportional hazard regression models for all variables reaching *p* < 0.05 by either of the tests to perform multivariate analysis. Other statistical differences were analyzed using the Chi-square test or Fisher’s exact test, and the Student’s *t*-test or Mann–Whitney *U*-test for categorical and continuous variables, respectively. Logistic regression analysis was used to identify predictive factors for responses leading to HPI eradication. All *p* values were two-sided, and a *p* < 0.05 indicated statistical significance. Statistical analysis was performed using the SPSS software version 24 (SPSS Inc., Chicago, IL, USA) and the “R” software version 3.4.1 (R Foundation for Statistical Computing, 2017).

## Results

### Patient baseline characteristics

A total of 203 patients with gastric MALT lymphoma were enrolled in this study, and all pathologic diagnoses were confirmed as low-grade MALT lymphoma. Their median age was 56 years (range, 21–87), and 120 patients (59.1%) were female. Most patients presented with epigastric pain (25.1%) or bloating (12.8%), but only 7.9% had B symptoms. The most frequent endoscopic findings were superficial erosions and ulcers (50.7%), often located in the distal two-thirds of the stomach (67.0%). A total of 74.4% cases of diagnosed gastric MALT lymphoma in this study were associated with HPI; the presence of HPI was confirmed by Warthin–Starry stain (30.6%), Giemsa stain (46.8%), PCR in the biopsy sample (22.6%), rapid urease test (29.1%), or combination of both 13C Urea breath test and *H. pylori* IgG positivity (6.6%). An HPI-negative status (25.6%) was confirmed by negative results in all HPI diagnostic methods. At the time of diagnosis, the MALT-IPI score was rated low in 142 (70.0%) patients, intermediate in 12 (5.9%) patients, and high in 49 (24.1%) patients. The clinical stage of most patients was stage I according to the Lugano and Paris staging system with T1–T4N0M0-1, B0 (86.7%), but 27 patients (13.4%) had stage ≥ II1, and 3.4% of the patients showed bone marrow involvement. The baseline characteristics of the enrolled patients are summarized in Table 1.

**Table 1:** Characteristics of gastric MALT lymphoma patients (total *n* = 203)

Characteristics	Values
Age, median (range)	56.0 (21.0–87.0) years
Sex	
Male	83 (40.9%)
Female	120 (59.1%)
Lactate dehydrogenase	364.0 (198.0–1640.0) IU/L
Elevated	27 (13.3%)
Normal	176 (86.7%)
ECOG performance status	
0–1	198 (97.5%)
2	5 (2.5%)
Clinical presentation	
No symptoms	112 (55.2%)
Epigastric pain	51 (25.1%)
Bloating	26 (12.8%)
Weight loss	11 (5.4%)
Nausea and vomiting	10 (4.9%)
GI bleeding	6 (3.0%)
B-symptoms	16 (7.9%)
Initial HPI positive†	151 (74.4%)
Diagnosis method	
Histology	
Warthin-Starry stain	38 (30.6%)
Giemsa stain	58 (46.8%)
PCR	28 (22.6%)
Rapid urease test (CLO test)	44 (29.1%)
13C Urea breath test with *H. pylori* IgG positive	10 (6.6%)
Initial HPI negative*	52 (25.6%)
Diagnostic location in gastroduodenoscopy	
Antrum	63 (31.0%)
Body	66 (32.5%)
High/middle/low body	20 (30.3%)/10 (15.2%)/36 (54.5%)
Antrum + low body	27 (13.3%)
Fundus	28 (13.8%)
Diffuse	19 (9.4%)
Anatomic location	
Proximal upper-third/multiple	67 (33.0%)
Distal two-thirds	136 (67.0%)
Endoscopic type	
Superficial	103 (50.7%)
Others §	100 (49.3%)
Invasion depth measured by EUS (*n* = 89)‡	
Mucosa	49 (55.1%)
Submucosa	25 (28.1%)
Muscularis propria to serosa	15 (16.8%)
MALT-IPI	
Low (0)	142 (70.0%)
Intermediate (1)	12 (5.9%)
High (2–3)	49 (24.1%)
Lugano stage and Paris TNMB stage**	
Localized stage	
Stage I (T1–4N0M0–1, B0)	176 (86.7%)
Stage II1 (T1–4N1M0–1, B0)	5 (2.5%)
Advanced stage	
Stage II2 (T1-4N2M0-1, B0)	1 (0.5%)
Stage IIE (T1–4N1M0–1, B0)	4 (2.0%)
Stage IV (T1–4N3M0–1B0, T1–4N0– 2M2B0 or B1)	17 (8.4%)
Bone marrow involvement	7 (3.4%)

*CLO*, *Campylobacte*r-like organism test; *ECOG*, Eastern Cooperative Oncology Group; *EUS*, endoscopic ultrasonography; *HPI*, *Helicobacter pylori* infection; *IU*, international unit; *MALT-IPI*, mucosa-associated lymphoid tissue lymphoma-International Prognostic Index; PCR, polymerase chain reaction

^†^Patients with at least one positive test result were defined as HPI-positive

^*^HPI-negative was confirmed by all negative tests in histology, serology, 13C urea breath test, and/or stool antigen test

^§^Endoscopically, other than superficial type tumors were classified as hypertrophic fold (*n* = 13), ulceroinfiltrative (*n* = 64), and ulcerofungating type (*n* = 23). *Ref.) Chin YJ, Chang DK, Lee KM, *et al. *(1996) Clinicopathologic study of primary gastric lymphoma of B-cell phenotype with special reference to low-grade B-cell lymphoma of MALT. Korean K Gastroenterol 31:463–476*

^‡^A total of 89 patients underwent EUS evaluation (Lugano stage I, *n* = 84). Two patients with Lugano stage IV showed muscularis propria invasion on EUS. Among the three patients with Lugano stage IIE, two patients also showed muscularis propria invasion, but one showed submucosal layer invasion on EUS

^**^A detailed contents of the Lugano stage-/Paris staging system are presented in Table S1

### H. pylori eradication and treatment responses in HPI-positive group patients

The clinical responses after first-line treatment are presented in Table 2. Among the 151 HPI-positive patients, 7 (4.6%) were diagnosed with Lugano stage IIE–IV and received radiotherapy or chemotherapy as first-line treatment. Therefore, 144 HPI-positive patients with stage I or II1–2 disease underwent *H. pylori* eradication, and 112 (77.8%) achieved CR. However, 15 (10.4%) and 17 (11.8%) patients showed endoscopic and histologic findings, respectively, indicative of either NC or rRD, consistent with resistance to HPI eradication treatment for up to 1 year after treatment. In addition, 8 of 112 (7.1%) patients who achieved CR presented with relapse during the follow-up period. One patient was diagnosed with myelodysplastic syndrome 2 years after achieving CR after HPI eradication.

**Table 2:** Treatment outcomes of gastric MALT lymphoma after initial treatment (total *n* = 203)

Variables	Values
HPI-positive, Stage I or II1 (*n* = 144)*	
First-line eradication	101 (49.8%)
Second-line eradication	43 (21.2%)
HPI eradication response rate	112/144 (77.8%)
Eradication failure	15 (10.4%)
* Treatment response*†	
Stable disease (NC)‡	15 (10.4%)
Partial remission (rRD) up to 12 months	17 (11.8%)
Complete remission (ChR and pMRD)§	112 (77.8%)
HPI-negative, Any stage (*n* = 59)	
Chemotherapy	27 (45.7%)
Radiotherapy	25 (42.4%)
Chemotherapy + consolidative radiotherapy	2 (3.4%)
Observation after EMR	5 (8.5%)
* Treatment response*	
Progressive disease	5 (8.5%)
Complete remission	54 (91.5%)

*CI*, confidence interval; *ChR*, complete histological remission; *EMR*, endoscopic mucosal resection; *HPI*, *Helicobacter pylori* infection; *NC*, no change; *pMRD*, probable minimal residual disease; *rRD*, responding residual disease

^*^Among 151 HPI-positive patients, 7 (4.6%) were diagnosed with Lugano stage IIE-IV and received chemotherapy

^†^Histological responses of post-treatment biopsies were classified using the GELA grading system

^‡^All patients with eradication failure showed no regression of gastric MALT lymphoma

^§^Complete remission after HPI eradication was defined as post-treatment biopsy histology showing ChR or pMRD in at least two subsequent follow-ups

The clinical characteristics of the non-responders and responders after HPI eradication are shown in Table 3. We observed that non-responders were associated with lesions in the proximal upper-third region or in multiple locations (46.9% vs. 21.4%, *p* = 0.004), and with deep submucosal invasion revealed by EUS (60.0% vs. 32.6%, *p* = 0.038). However, other characteristics such as age, sex, superficial macroscopic findings, Lugano stage, or MALT-IPI risk did not differ significantly between the two groups. Multivariate logistic regression analysis showed that lesions in the proximal two-thirds or multiple locations were predictors of HPI eradication treatment failure (odds ratio [OR] 3.235, 95% confidence interval [CI], 1.413–7.406, *p* = 0.005). In 66 patients who underwent EUS, submucosa or deeper invasion was a predictor of resistance to HPI eradication (OR 3.100, 95% CI, 1.046–9.187, *p* = 0.041). A comparison of clinical characteristics and HPI eradication response according to the level of gastric layer involvement in EUS is presented in Online Resource 3.

**Table 3:** Predictive factors for HPI eradication resistance as determined by logistic regression

Variables	Non-responder* (*n* = 32)	Responder (*n* = 112)	*p*-value	All patients (*n* = 144)		Selected patients (*n* = 66)†	
				OR (95% CI)	*p*-value	OR (95% CI)	*p*-value
Age ≥ 60	8 (25.0%)	37 (33.0%)	0.387	1.567 (0.608–4.041)	0.353	1.032 (0.232–4.597)	0.967
Male	16 (50.0%)	41 (36.6%)	0.172	1.908 (0.836–4.352)	0.125	1.664 (0.541–5.116)	0.374
Proximal upper-third/multiple	15 (46.9%)	24 (21.4%)	*0.004*	3.235 (1.413–7.406)	*0.005*	2.628 (0.823–8.395)	0.103
Superficial type	15 (46.9%)	63 (56.2%)	0.348	0.835 (0.362–1.928)	0.673	0.871 (0.281–2.700)	0.811
Lugano stage I	31 (96.9%)	110 (98.2%)	0.532	0.950 (0.066–13.62)	0.950	-	-
MALT IPI low risk	26 (81.3%)	89 (79.5%)	0.824	0.842 (0.268–2.643)	0.768	0.968 (0.235–3.982)	0.964
EUS (≥ SM) (*n* = 66)	12 (60.0%)	15 (32.6%)	*0.038*			3.100 (1.046–9.187)	*0.041*

*OR*, odds ratio; *CI*, confidence interval; *EUS*, endoscopic ultrasonography; *HPI*, *Helicobacter pylori* infection; *MALT-IPI*, mucosa-associated lymphoid tissue lymphoma-International Prognostic Index; *SM*, submucosa

^*^Non-responders were defined as delayed responders (rRD) who did not achieve CR after 1-year follow-up or patients with NC, eradication failure after 2nd line therapy, macroscopic SD/PD, or rRD with PR in macroscopic findings up to 1 year

^†^Patients who underwent EUS workup

### Treatment and responses of HPI-negative group patients

The 59 patients diagnosed with an absence of HPI in any Lugano stage underwent chemotherapy (45.7%), radiation therapy (42.4%), or both (3.4%). Fifteen (8.5%) patients with a single small lesion that did not invade deeper than the mucosa underwent only EMR and achieved long-term CR. Two patients were treated with chemotherapy along with consolidative radiotherapy and achieved long-term CR without relapse. Eventually, 54 (91.5%) patients achieved CR, whereas five patients showed PD on CT and 18F-FDG PET-CT. The first-line treatment response and adverse events associated with radiotherapy and chemotherapy are presented in Table 4. Among the 25 radiotherapy-treated patients, except one with stage II1 96.0% (*n* = 24) were Lugano stage I, and 68.0% (*n* = 17) were at low risk of MALT-IPI. Twenty-seven patients received chemotherapy, and 63.0% (*n* = 17), 14.8% (*n* = 4), 3.7% (*n* = 1), and 18.5% (*n* = 5) of the patients were at Lugano stage IV, IIE, II2, and I, respectively. Five stage I patients were t(11;18)/*API2-MALT1* positive, and one stage II2 patient refused to receive radiotherapy.

**Table 4:** Treatment outcomes and adverse events in HPI-negative group patients (*n* = 52) †

Category	First-line radiotherapy (*n* = 25)	First-line chemotherapy (*n* = 2 7)	*p-*value
Treatment modalities	3060 cGy dose, 17 fraction Whole-stomach irradiation	Eight cycles of R-CVP	-
			-
*Clinical characteristics*			
Age			0.554
≥ 60 years (*n* = 29)	15 (60.0%)	14 (51.9%)	
< 60 years (*n* = 23)	10 (40.0%)	13 (48.1%)	
Sex			0.250
Male (*n* = 23)	9 (36.0%)	14 (51.9%)	
Female (*n* = 29)	16 (64.0%)	13 (48.1%)	
Dominant site of lesion			0.797
Proximal upper-third/multiple (*n* = 39)	13 (52.0%)	15 (55.6%)	
Distal two-thirds (*n* = 105)	12 (48.0%)	12 (44.4%)	
Endoscopic type			0.174
Superficial (*n* = 78)	13 (52.0%)	19 (70.4%)	
Others (*n* = 66)	12 (48.0%)	8 (29.6%)	
Lugano stage			< *0.001*
Stage I (*n* = 29)	24 (96.0%)	5 (18.5%) ‡	
Stage II1–2 (*n* = 2)	1 (4.0%)	1 (3.7%)	
Stage IIE (*n* = 4)	0	4 (14.8%)	
Stage IV (*n* = 17)	0	17 (63.0%)	
MALT-IPI			< *0.001*
Low (*n* = 21)	17 (68.0%)	4 (14.8%)	
Intermediate to high (*n* = 31)	8 (32.0%)	23 (85.2%)	
ECOG performance status			< *0.001*
0–1 (*n* = 47)	25 (100%)	22 (81.5%)	
2 (*n* = 5)	0	5 (18.5%)	
*Treatment outcomes*			
CR	25 (100%)	22 (81.5%)	0.052
PD	0	5 (18.5%)	0.165
*Adverse events*			
AE grade (NCI-CTC-AE ver. 5.0)	Gastric discomfort (*n* = 4, 16.0%) Grade III–IV (*n* = 0)	Neutropenia (*n* = 19, 70.4%) Grade III–IV (*n* = 11, 40.7%)	-
	Nausea (*n* = 3, 12.0%) Grade III–IV (*n* = 1, 4.0%)	Anemia (*n* = 12, 44.5%) Grade III–IV (*n* = 4, 14.8%)	-
	-	Thrombocytopenia (*n* = 6, 22.2%) Grade III–IV (*n* = 2, 7.4%)	-
	-	Peripheral neuropathy (*n* = 7, 25.9%) Grade III–IV (*n* = 0)	-
	-	Infection (*n* = 3, 11.1%) Grade III–IV (*n* = 1, 3.7%)	-

*CR*, complete remission; *EUS*, endoscopic ultrasonography; *HPI*, *Helicobacter pylori* infection; *MALT-IPI*, mucosa-associated lymphoid tissue lymphoma-International Prognostic Index; *PD*, progression of disease

^†^Two patients treated with chemotherapy with consolidative radiotherapy and five patients treated with EMR were not included in this analysis. All patients achieved long-term CR

^‡^Five patients diagnosed with low-grade gastric MALT lymphoma showed t(11;18)/*API2-MALT1* positive

All HPI-negative patients who received first-line radiotherapy achieved CR (100%), and only one patient relapsed. However, 22 of 27 first-line chemotherapy-treated patients achieved CR (81.5%), five showed PD, and seven relapsed. Three relapsed patients showed the high-grade transformation of MALT lymphoma, which pathologically consisted of diffuse large B-cell lymphoma. During eight standard cycles of R-CVP chemotherapy treatment-related adverse events were mainly hematologic, with grade III–IV neutropenia, anemia, and thrombocytopenia in 40.7%, 14.8%, and 7.4% of the patients, respectively. All patients were treated with filgrastim injection as neutropenia prophylaxis, and three of them experienced febrile neutropenia without evidence of infection. Peripheral neuropathy was frequently reported as a non-hematologic adverse event (25.9%), but most cases were controlled well by oral gabapentin and none were in Grades III–IV. Three patients experienced non-neutropenic fever with bronchopneumonia, herpes zoster, and septic shock due to proctitis. The detailed treatment outcomes of patients with gastric MALT lymphoma who received second-line salvage treatments are shown in Online Resource 4.

### Clinical outcomes and prognostic factor analysis

**Fig. 2:**
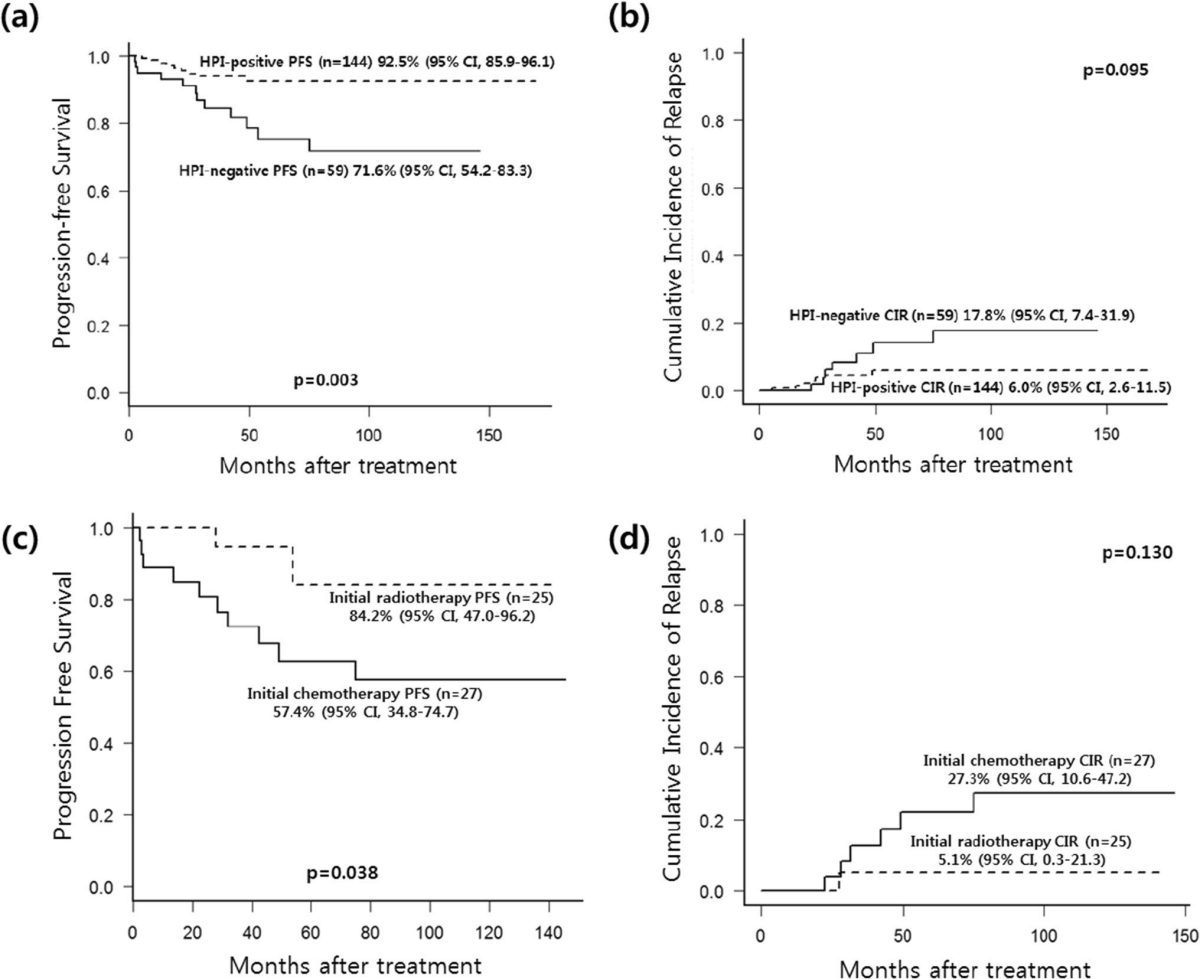
Kaplan–Meier survival curves according to treatment modality in gastric MALT lymphoma. Kaplan–Meier survival curves of PFS (**a**) and CIR (**b**) according to HPI-positive versus HPI-negative gastric MALT lymphoma patients. The PFS (**c**) and CIR (**d**) curves of patients initially treated with radiotherapy versus chemotherapy in gastric MALT lymphoma

In the median follow-up period of 51.0 months (range, 3.0–230.4), CIR was 10.9% (95% CI, 6.3–16.8), TRM was 1.5% (95% CI, 0.4–4.2), OS was 98.5% (95% CI, 93.9–99.6), and PFS was 86.1% (95% CI, 79.0–91.0). A significant difference in PFS between HPI-positive gastric MALT lymphoma patients treated with *H. pylori* eradication and HPI-negative patients who underwent radiotherapy, chemotherapy, chemoradiotherapy, or EMR (92.5% vs. 71.6%, *p* = 0.003) was identified. The HPI-negative group also showed a trend of elevated CIR (6.0% vs. 17.8%, *p* = 0.095). Furthermore, comparing the PFS and CIR of each treatment modality in the HPI-negative group revealed that radiotherapy was associated with significantly better PFS (84.2% vs. 57.4%, *p* = 0.038) and a trend of lesser CIR (5.1% vs. 27.3%, *p* = 0.130) compared to chemotherapy. Five HPI-negative patients underwent EMR, and two of the chemoradiotherapy-treated patients did not experience relapse or non-relapse mortality and were still alive. The clinical outcomes between different treatment modalities are shown in Fig. 2.

Univariate (Online Resource 5) and multivariate (Table 5) analyses revealed that HPI negativity was significantly correlated with poor PFS (HR 3.281, 95% CI, 1.393–7.725, *p* = 0.006), and lesions in the proximal upper-third region or in multiple locations were significantly correlated with higher CIR (HR 3.442, 95% CI, 1.246–9.509, *p* = 0.007). Furthermore, lesions in the proximal upper-third or in multiple locations were identified as significant factors for poor PFS (84.0% vs. 95.6%, *p* = 0.043) and higher CIR (16.0% vs. 3.4%, *p* = 0.018) in the HPI-treated subgroup in the univariate analysis (Online Resource 6).

**Table 5:** Multivariate analysis for survival outcomes in primary gastric MALT lymphoma patients (Entire cohort, *n* = 203) †

Variables	HR (95% CI)	*p*-values
PFS		
Male	2.274 (0.939–5.505)	0.069
Non-superficial endoscopic type	2.071 (0.796–5.389)	0.136
HPI negative	3.281 (1.393–7.725)	*0.006*
CIR		
Proximal upper-third/multiple location	3.442 (1.246–9.509)	*0.007*
HPI negative	2.305 (0.837–6.351)	0.110

*CI*, confidence interval; *CIR*, cumulative incidence of relapse; *HR*, hazard ratio; *HPI*, *Helicobacter pylori* infection; PFS, progression-free survival

^†^Multivariate modes were derived using stepwise selection among candidate variables with the Wald test for overall *p*-value for factors with > 2 levels and *p*-value < 0.05, to warrant inclusion in the model. Furthermore, HPI status and Lugano stage variables showed a significant correlation, and we selected HPI status as a multivariate analysis variable

## Discussion

The real-world clinical outcomes of gastric MALT lymphoma treated with different therapeutic modalities were presented in this long-term, retrospective, single-center study with sufficient patients regardless of the stage. It is currently widely accepted that eradication of HPI has become a standard treatment for gastric MALT lymphoma based on chronic HPI, which provides antigen stimulus to gastric MALT lymphoma through the clonal expansion of lymphoid cells [22]. HPI-positive patients with localized stage cancers who achieved long-term CR solely with eradication were supported by several prior studies presenting a high remission rate of approximately 80% [1, 8, 9]. In our study, the HPI eradication CR rate was 77.8% in 144 HPI-positive stage I or II1 patients, consistent with recent large cohort study results. Most of these patients achieved CR in less than 12 months (range, 3.8–13.2). For a possible delay in achieving histologic remission of lymphoma, we also adopted the watch-and-wait strategy for at least 12 months according to the current European Society of Medical Oncology (ESMO) guidelines [8, 10].

However, the following patients should be considered for different treatment options: diagnosed HPI-negative, not achieving CR in either endoscopic or histologic findings after eradication, presenting positive for t(11;18)(p21;p21)/*API2-MALT1*, relapse after achieving CR, or with advanced stage of II2, IIE or IV. Due to the uncertainty and known low efficacy of eradication treatment in HPI-negative patients, we did not perform eradication for HPI-negative patients. Instead, stage I or II1 HPI-negative patients showed an excellent treatment response with a 100% CR rate after radiotherapy. A recently published multicenter cohort study performed in Japan showed a 13.6% CR rate, and retrospective pooled analysis including 110 patients with HPI-negative gastric MALT lymphoma eradication revealed a 15.5% CR rate [1, 23]. These results suggest that the efficacy of eradication treatment in HPI-negative patients has not yet been determined and is questionable. Nevertheless, the EGILS group consensus and ESMO guidelines recommend that eradication should be considered before radiotherapy or chemotherapy, based on the evidence of occasional lymphoma response probably due to a false-negative test or infection with other *Helicobacter* species [10, 19]. Because of the indolent nature of gastric MALT lymphoma and guideline recommendations, clinicians may consider eradication treatment in HPI-negative stage I or II1 patients and carefully watch and wait with a routine endoscopic assessment. If patients with NC or rRD persist for 2–3 months after antibiotic administration, clinicians may recommend subsequent radiation therapy [19].

Radiation is mainly considered in stage I or II1, either as initial therapy for HPI-negative patients or for HPI-positive patients that experienced eradication failure [19, 20]. The clinical outcomes of radiotherapy in HPI-negative (*n* = 27) or eradication failure patients (*n* = 33) were excellent, presenting a 100% CR rate and a notable safety profile with only one patient experiencing relapse. We also used the “watch-and-wait” strategy for 12 months without further treatment for delayed responders in radiotherapy, which was previously presented in our institute [20]. A recent retrospective study also shows excellent radiotherapy outcomes in HPI-negative gastric MALT lymphoma patients, with a 5- and 10-year OS of 94% and 79%, respectively, and with only 9.6% local failures [24]. In our data, the OS, PFS, and CIR of first-line radiotherapy-treated patients (*n* = 25) with HPI-negative stage I or II1-2 were 100%, 84.2%, and 5.1%, respectively, at the 19-year follow-up. Systemic chemotherapy is considered only for first-line treatment in advanced-stage patients or for relapsed/refractory disease after prior treatments, including HPI eradication and radiotherapy. In 27 first-line R-CVP chemotherapy-treated HPI-negative stage II2, IIE, or IV patients, CR was observed in 81.5%, relapse after CR in 22.2%, OS in 90.7%, and PFS in 57.4% of the patients at the 19-years follow-up. Our results showed inferior outcomes with considerable hematologic adverse events compared to previous studies [19, 25, 26]. Thus, further studies utilizing different chemotherapy regimens are necessary to confirm the optimal treatment modalities for advanced-stage or relapsed/refractory gastric MALT lymphoma.

Several known predictive factors for resistance to HPI eradication have been identified, including male sex, negative HPI status, proximal gastric locations, stage II1 or advanced, depth of invasion by EUS beyond the submucosal layer, non-superficial endoscopic type, and t(11;18)(p21;p21)/*API2-MALT1* translocation positive [1]. Our results also showed that lesions in the proximal upper-third or in multiple locations and invasion depth to the submucosa or deeper in EUS were associated with poor response to eradication. However, the endoscopic type did not show a difference between responders and non-responders. As most stage II1–2, IIE, or IV patients underwent radiation or chemotherapy regardless of HPI status, we could not identify advanced stage or HPI-negative status as predictive factors of eradication response. Furthermore, we were unable to examine the status of the t(11;18)(p21;p21)/*API2-MALT1* translocation in all enrolled patients, which is a well-known risk factor for predicting eradication response. Primarily due to financial constraints, only a proportion of patients agreed to undergo the t(11;18)(p21;p21)/*API2-MALT1* translocation FISH test. For a similar reason, EUS was also examined in a limited number of patients, raising the possibility of bias in the study. Given the low incidence of gastric MALT lymphoma, selection bias is also possible due to the small sample size of each treatment group, especially in stages II1–2 or IIE.

With respect to prognostic factors for survival outcomes, HPI-negative gastric MALT lymphoma regardless of stage showed poor PFS (HR 3.281, 95% CI, 1.393–7.725, *p* = 0.006), and disease in proximal upper-third or multiple locations was closely related to higher CIR (HR 3.442, 95% CI, 1.246–9.509, *p* = 0.007). It is interesting that location also significantly affected the eradication response. Although gastric MALT lymphoma progresses slowly and prognosis tends to be favorable, HPI-negative, advanced stage, or eradication-resistant patients showed relatively poor clinical outcomes with potential risk for high-grade transformation, and clear treatment guidelines are yet to be established.

In summary, our results confirm that HPI eradication treatment should be the first-line treatment modality for localized stage HPI-positive gastric MALT lymphoma, with a “watch-and-wait” strategy for delayed responders for at least 1 year. Radiotherapy is recommended for patients with localized HPI-negative status or with eradication failure, which may also indicate a watch-and-wait approach. Chemotherapy is the treatment of choice for advanced-stage patients regardless of HPI status, and large multicenter studies to elucidate appropriate chemotherapy regimens, including novel agents, may help improve treatment outcomes. Furthermore, due to the considerable relapse rate compared to excellent survival outcomes, long-term regular monitoring is required for all patients.

## Supplementary Information

Below is the link to the electronic supplementary material.

Supplementary file1 (DOCX 48 KB)

## Data Availability

The data that support the findings of this study are available upon request from the corresponding author. The data are not publicly available because of privacy or ethical restrictions.

## References

[1] Nakamura S, Sugiyama T, Matsumoto T, Iijima K, Ono S, Tajika M, Tari A, Kitadai Y, Matsumoto H, Nagaya T, Kamoshida T, Watanabe N, Chiba T, Origasa H, Asaka M, Japan GAST Study Group (2012). Long-term clinical outcome of gastric MALT lymphoma after eradication of *Helicobacter pylori*: a multicentre cohort follow-up study of 420 patients in Japan. Gut.

[2] Isaacson PG, Du MQ (2005). Gastrointestinal lymphoma: where morphology meets molecular biology. J Pathol.

[3] Park G, Kang CS (2012). Pathophysiology of gastric MALT lymphoma. Korean J Med.

[4] Choi MK, Kim GH (2011). Diagnosis and treatment of gastric MALT lymphoma. Korean J Gastroenterol.

[5] Weber DM, Dimopoulos MA, Anandu DP, Pugh WC, Steinbach G (1994). Regression of gastric lymphoma of mucosa-associated lymphoid tissue with antibiotic therapy for Helicobacter pylori. Gastroenterology.

[6] Wotherspoon AC, Doglioni C, Diss TC, Pan L, Moschini A, de Boni M, Isaacson PG (1993). Regression of primary low-grade B-cell gastric lymphoma of mucosa-associated lymphoid tissue type after eradication of Helicobacter pylori. Lancet.

[7] Hussell T, Isaacson PG, Crabtree JE, Spencer J (1993). The response of cells from low-grade B-cell gastric lymphomas of mucosa-associated lymphoid tissue to Helicobacter pylori. Lancet.

[8] Matysiak-Budnik T, Fabiani B, Hennequin C, Thieblemont C, Malamut G, Cadiot G, Bouché O, Ruskoné-Fourmestraux A (2018). Gastrointestinal lymphomas: French Intergroup clinical practice recommendations for diagnosis, treatment and follow-up (SNFGE, FFCD, GERCOR, UNICANCER, SFCD, SFED, SFRO, SFH). Dig Liver Dis.

[9] Stathis A, Chini C, Bertoni F, Proserpio I, Capella C, Mazzucchelli L, Pedrinis E, Cavalli F, Pinotti G, Zucca E (2009). Long-term outcome following Helicobacter pylori eradication in a retrospective study of 105 patients with localized gastric marginal zone B-cell lymphoma of MALT type. Ann Oncol.

[10] Zucca E, Copie-Bergman C, Ricardi U, Thieblemont C, Raderer M, Ladetto M, ESMO Guidelines Working Group (2013) Gastric marginal zone lymphoma of MALT type: ESMO clinical practice guidelines for diagnosis, treatment and follow-up. Ann Oncol 24 Suppl 6:vi144–vi148. 10.1093/annonc/mdt343 10.1093/annonc/mdt343 24078657

[11] Ye H, Liu H, Raderer M, Chott A, Ruskone-Fourmestraux A, Wotherspoon A, Dyer MJ, Chuang SS, Dogan A, Isaacson PG, Du MQ (2003). High incidence of t(11;18)(q21;q21) in Helicobacter pylori–negative gastric MALT lymphoma. Blood.

[12] Swerdlow SH, Campo E, Harris NL (2008). WHO classification of tumours of haematopoietic and lymphoid tissues.

[13] Hummel M, Oeschger S, Barth TFE, Loddenkemper C, Cogliatti SB, Marx A, Wacker HH, Feller AC, Bernd HW, Hansmann ML, Stein H, Möller P (2006). Wotherspoon criteria combined with B cell clonality analysis by advanced polymerase chain reaction technology discriminates covert gastric marginal zone lymphoma from chronic gastritis. Gut.

[14] Song ISCK, Kim CY (1998). Clinicopathologic study of primary gastric lymphoma of B-cell lymphoma of MALT. Korean J Gastroenterol.

[15] Thieblemont C, Cascione L, Conconi A, Kiesewetter B, Raderer M, Gaidano G, Martelli M, Laszlo D, Coiffier B, Lopez Guillermo A, Torri V, Cavalli F, Johnson PW, Zucca E (2017). A MALT lymphoma prognostic index. Blood.

[16] Musshoff K (1977). Clinical staging classification of non-Hodgkin’s lymphomas (author’s transl). Strahlentherapie.

[17] Ruskoné-Fourmestraux A, Dragosics B, Morgner A, Wotherspoon A, De Jong D (2003). Paris staging system for primary gastrointestinal lymphomas. Gut.

[18] Rohatiner A, d’Amore F, Coiffier B, Crowther D, Gospodarowicz M, Isaacson P, Lister TA, Norton A, Salem P, Shipp M (1994). Report on a workshop convened to discuss the pathological and staging classifications of gastrointestinal tract lymphoma. Ann Oncol.

[19] Ruskoné-Fourmestraux A, Fischbach W, Aleman BM, Boot H, Du MQ, Megraud F, Montalban C, Raderer M, Savio A, Wotherspoon A, EGILS group,  (2011). EGILS consensus report EGILS consensus report. Gastric extranodal marginal zone B-cell lymphoma of MALT. Gut.

[20] Choi KH, Lee HH, Jung SE, Park KS, O JH, Jeon YW, Choi BO, Cho SG (2020). Analysis of the response time to involved-field radiotherapy in primary gastrointestinal low-grade B-cell lymphoma. Radiat Oncol.

[21] Copie-Bergman C, Wotherspoon AC, Capella C, Motta T, Pedrinis E, Pileri SA, Bertoni F, Conconi A, Zucca E, Ponzoni M, Ferreri AJ (2013). Gela histological scoring system for post-treatment biopsies of patients with gastric MALT lymphoma is feasible and reliable in routine practice. Br J Haematol.

[22] Owens SR, Smith LB (2011) Molecular aspects of H. pylori-related MALT lymphoma. Patholog Res Int 2011:193149. 10.4061/2011/193149 10.4061/2011/193149 PMC3034981 21318155

[23] Zullo A, Hassan C, Ridola L, De Francesco V, Rossi L, Tomao S, Vaira D, Genta RM (2013). Eradication therapy in Helicobacter pylori-negative, gastric low-grade mucosa–associated lymphoid tissue lymphoma patients: a systematic review. J Clin Gastroenterol.

[24] Yahalom J, Xu AJ, Noy A, Lobaugh S, Chelius M, Chau K, Portlock C, Hajj C, Imber BS, Straus DJ, Moskowitz CH, Coleman M, Zelenetz AD, Zhang Z, Dogan A (2021). Involved-site radiotherapy for Helicobacter pylori–independent gastric MALT lymphoma: 26 years of experience with 178 patients. Blood Adv.

[25] Cencini E, Fabbri A, Lauria F, Bocchia M (2018). Long-term efficacy and toxicity of rituximab plus fludarabine and mitoxantrone (R-FM) for gastric marginal zone lymphoma: a single-center experience and literature review. Ann Hematol.

[26] Zucca E, Conconi A, Laszlo D, López-Guillermo A, Bouabdallah R, Coiffier B, Sebban C, Jardin F, Vitolo U, Morschhauser F, Pileri SA, Copie-Bergman C, Campo E, Jack A, Floriani I, Johnson P, Martelli M, Cavalli F, Martinelli G, Thieblemont C (2013). Addition of rituximab to chlorambucil produces superior event-free survival in the treatment of patients with extranodal marginal-zone B-cell lymphoma: 5-year analysis of the IELSG-19 randomized study. J Clin Oncol.

