# Supplementary material for: Long-term clinical outcomes of gastric mucosa-associated lymphoid tissue lymphoma in real-world experience
Source: Ann Hematol. 2023 Feb 25;102(4):877–88. doi: 10.1007/s00277-023-05130-8 (PMC9998564; doi:10.1007/s00277-023-05130-8)
Supplement: Supplementary file 1 — Supplementary file1 (DOCX 48 KB) [file 277_2023_5130_MOESM1_ESM.docx]

**Supplementary Information**

**Title:** Long-Term Clinical Outcomes of Gastric Mucosa-associated Lymphoid Tissue Lymphoma in Real-world Experience

**Journal name:** *Annals of Hematology*

Gi-June Min^1^, Donghoon Kang^6^, Han Hee Lee^7^, Seung-Jun Kim^8^, Tong Yoon Kim^1^, Young-Woo Jeon^2^, Joo Hyun O^3^, Byung-Ock Choi^4^, Gyeongsin Park^5^, and Seok-Goo Cho^1^*

^1^Department of Hematology, Catholic University Lymphoma Group, Seoul St. Mary's Hospital, College of Medicine, The Catholic University of Korea, Banpo-daero 222, Seocho-Gu, Seoul, Republic of Korea

^2^Department of Hematology, Catholic University Lymphoma Group, Yeouido St. Mary's Hospital, College of Medicine, The Catholic University of Korea, 10^,^ 63-ro, Yeongdeungpo-gu, Seoul, Republic of Korea

^3^Department of Nuclear Medicine, Catholic University Lymphoma Group, Seoul St. Mary's Hospital, College of Medicine, The Catholic University of Korea, Banpo-daero 222, Seocho-Gu, Seoul, Republic of Korea

^4^Department of Radiation Oncology, Catholic University Lymphoma Group, Seoul St. Mary's Hospital, College of Medicine, The Catholic University of Korea, Banpo-daero 222, Seocho-Gu, Seoul, Republic of Korea

^5^Department of Hospital Pathology, Catholic University Lymphoma Group, Seoul St. Mary's Hospital, College of Medicine, The Catholic University of Korea, Banpo-daero 222, Seocho-Gu, Seoul, Republic of Korea

^6^Department of Gastroenterology, Seoul St. Mary's Hospital, College of Medicine, The Catholic University of Korea, Banpo-daero 222, Seocho-Gu, Seoul, Republic of Korea

^7^Department of Gastroenterology, Yeouido St. Mary's Hospital, College of Medicine, The Catholic University of Korea, 10^,^ 63-ro, Yeongdeungpo-gu, Seoul, Republic of Korea

^8^Department of Gastroenterology, H+ Yangji Hospital, 1636, Nambusunhwan-ro, Gwanakgu, Seoul, Republic of Korea

*** Corresponding author:**

Seok-Goo Cho

E-mail: chosg@catholic.ac.kr

**Online Resource 1. The staging system for primary gastric MALT lymphoma**

| **Lugano stage**† | **Lugano concept** | **Modified Ann-Arbor**‡ | **Paris TNMB stage**§ | **Extent of lymphoma** |
| --- | --- | --- | --- | --- |
| **Stage I** | Confined to the GI tract: single primary or multiple, non-contiguous | **IE1** | **T1N0M0–1B0** | Mucosa, submucosa |
|  |  | **IE2** | **T2N0M0–1B0** | Muscularis propria, submucosa |
|  |  | **IE2** | **T3N0M0–1B0** | Serosa penetration |
| **Stage II1** | Extending into abdomen | **IIE1** | **T1–4N1M0–1B0** | Regional LN |
| **Stage II2** |  | **IIE2** | **T1–4N2M0–1B0** | Intra-abdominal distant LNs |
| **Stage IIE** | Penetration of serosa to involve adjacent organs or tissue | **IE2** | **T1–4N1M0–1B0** | Invasion of neighboring organ |
| **Stage IV** | Disseminated extranodal involvement of concomitant supra-diaphragmatic nodal involvement | **IIIE** | **T1–4N3M0–1B0** | Extra-abdominal LN |
|  |  | **IVE** | **T1–4N0–2M2B0** | Diffuse/disseminated spread |
|  |  | **IVE** | **T1–4N0–2M0–1B1** | BM involvement |

GI, gastrointestinal; T, describes the gastric wall infiltration; N describes the regional lymph node involvement; M describes distant dissemination; B describes the bone marrow assessment

† Rohatiner A, d’Amore F, Coiffier B et al (1994) Report on a workshop convened to discuss the pathological and staging classifications of gastrointestinal tract lymphoma. Ann Oncol 5:397–400. https://doi.org/10.1093/oxfordjournals.annonc.a058869 We analyze patients mainly with the Lugano stage.

‡ Wotherspoon AC, Doglioni C, Diss TC, et al (1993) Regression of primary low-grade B-cell gastric lymphoma of mucosa-associated lymphoid tissue type after eradication of Helicobacter pylori. Lancet 342:575–577. https://doi.org/10.1016/0140-6736(93)91409-f

§ Ruskone-Fourmestraux A, Dragosics B, Morgner A et al (2003) Paris staging system for primary gastrointestinal lymphomas. Gut 52:912–913. https://doi.org/10.1136/gut.52.6.912

**Online Resource 2. The post-treatment response evaluation for primary gastric MALT lymphoma** †

| **GELA grading system** ‡ | **Histology** | **Clinical response** |
| --- | --- | --- |
| **Complete histological response (CR)** | Entire disappearance of the lymphoid infiltrate with only scattered plasma cells and small lymphocytes.  Regressive stromal change with fibrosis and separation of glands can be seen. | **Complete remission** |
| **Probable minimal residual disease (pMRD)** | The presence of small lymphoid aggregates, usually at the base of the lamina propria, associated with aggressive stromal changes. | **Complete remission** |
| **Responding residual disease (rRD)** | Overt residual lymphoma with diffuse or nodular infiltration of B-cell neoplasm, but with clear evidence of regressive stromal changes characterized by fibrosis and an empty lamina propria. | **Partial remission** |
| **No change (NC)** | Persistence of overt lymphoma identical to that seen at diagnosis with no morphological features suggesting a response to treatment, such as stromal fibrosis. | **Stable disease or**  **progressive disease** |

† Ruskone-Fourmestraux A, Fischbach W, Aleman BM et al (2011) EGILS consensus report. Gastric extranodal marginal zone B-cell lymphoma of MALT. Gut 60:747–758. https://doi.org/ 10.1136/gut.2010.224949

‡ Copie-Bergman C, Gaulard P, Lavergne-Slove A, et al (2003) Proposal for a new histological grading system for post-treatment evaluation of gastric MALT lymphoma. Gut 52:1656. https://doi.org/ 10.1136/gut.52.11.1656

**Online Resource 3. Comparison of clinical characteristics and HPI eradication response according to the level of gastric layer involvement in EUS (n=66)**

| **Category** | **Mucosa invasion (n=39)** | **Submucosa or deeper layer invasion (n=27)** | ***p-*value** |
| --- | --- | --- | --- |
| ***Clinical characteristics*** |  |  |  |
| **Age** |  |  | 0.091 |
| ≥60 years (n=17) | 13 (33.3%) | 4 (14.8%) |  |
| <60 years (n=49) | 26 (66.7%) | 23 (85.2%) |  |
| **Sex** |  |  | 0.566 |
| Male (n=29) | 16 (41.0%) | 13 (48.1%) |  |
| Female (n=37) | 23 (59.0%) | 14 (51.9%) |  |
| **Dominant site of lesion** |  |  | 0.218 |
| Proximal upper-third/multiple (n=19) | 9 (23.1%) | 10 (37.0%) |  |
| Distal two-thirds (n=47) | 30 (76.9%) | 17 (63.0%) |  |
| **Endoscopic type** |  |  | 0.339 |
| Superficial (n=34) | 22 (56.4%) | 12 (44.4%) |  |
| Others (n=32) | 17 (43.6%) | 15 (55.6%) |  |
| **MALT-IPI** |  |  | 0.497 |
| Low (n=51) | 29 (74.4%) | 22 (81.5%) |  |
| Intermediate to high (n=15) | 10 (25.6%) | 5 (18.5%) |  |
| ***Clinical outcomes*** |  |  |  |
| **HPI eradication response** |  |  |  |
| Complete remission (ChR and pMRD) (n=46) | 31 (79.5%) | 15 (55.6%) | *0.038* |
| Partial remission (rRD) up to 12 months (n=9) | 3 (7.7%) | 6 (22.2%) | 0.083 |
| Stable disease (NC) (n=11) | 5 (12.8%) | 6 (22.2%) | 0.336 |
| **1^st^ Relapse after achieving Complete remission** | 0 (0%) | 1 (3.7%) | 0.409 |
| **Receiving salvage treatment rate** | 8 (20.5%) | 13 (48.1%) | *0.018* |

CR, complete remission; EUS, endoscopic ultrasonography; HPI, *Helicobacter pylori* infection; MALT-IPI, mucosa-associated lymphoid tissue lymphoma-International Prognostic Index

**Online Resource 4. Clinical outcomes of gastric MALT lymphoma who received second-line treatment (total n=203)**

| **First-line treatment** | **Number of patients** | **2^nd^ line treatment** | **Response to**  **2^nd^ line treatment** | **Clinical course** |
| --- | --- | --- | --- | --- |
| **HPI-positive, Stage IE or II1~~-2~~ (n=144) *** | | | | |
| Stable disease (NC) with eradication failure | 15 | Radiotherapy (15) | CR (15) | All achieved long-term CR. |
| Partial remission (rRD) up to 12 months | 17 | Radiotherapy (13) | CR (13) | All achieved long-term CR. |
|  |  | Chemotherapy (4) ** | CR (4) | One patient experienced short-term relapse (7 mo.) after #8 R-CVP chemotherapy and achieved CR after salvage #6 CHOP. The others achieved long-term CR. |
| Relapsed after CR | 8 | Radiotherapy (4) | CR (4) | All achieved long-term CR. |
|  |  | Chemotherapy (2) † | CR (2) |  |
|  |  | Chemoradiotherapy (2) | CR (2) |  |
| **HPI-negative, any stage** **(n=59)** | | | | |
| Progressive disease after chemotherapy | 5 | Chemotherapy (5) | CR (2) | All achieved long-term CR. |
|  |  |  | Refractory (3) ‡ | Among three refractory patients, one patient expired after autologous HSCT because of septic shock. |
| Relapsed after CR | 8 | Radiotherapy (1) | CR (1) | A patient achieved long-term CR. |
|  |  | Chemotherapy (7) § | CR (6) | One Stage IV patient experienced a relapse as a high-grade transformation of MALT lymphoma after R-CVP chemotherapy and expired during salvage chemotherapy because of septic shock. The others achieved long-term CR. |

CI, confidence interval; CR, complete remission; EMR, endoscopic mucosal resection; HSCT, hematopoietic stem cell transplantation

* Among 151 HPI-positive patients, 7 (4.6%) were diagnosed with Lugano stage II2, IIE, and IV and received chemotherapy.

** Four patients had a preference for systemic chemotherapy rather than radiotherapy after HPI eradication treatment failure.

†One patient was diagnosed with relapse with an isolated pulmonary lesion, and the other with a thyroid lesion.

§Three patients relapsed as a high-grade transformation of MALT lymphoma, which pathologically consists of diffuse large B-cell lymphoma and is treated with R-CHOP.

**Online Resource 5. Univariate analysis of survival outcomes in primary gastric MALT lymphoma patients (Entire cohort, n=203)** †

| Variables | OS | *p-*value | PFS | *p-*value | CIR | *p-*value | DRM | *p-*value |
| --- | --- | --- | --- | --- | --- | --- | --- | --- |
| Age (years) |  | 0.066 |  | 0.595 |  | 0.571 |  | 0.300 |
| <60 (n=126) | 100% |  | 86.6% (76.8–92.5) |  | 12.6% (6.3–21.2) |  | 0.8% (0.1–4.0) |  |
| ≥60 (n=77) | 96.0% (84.2–99.0) |  | 85.0% (72.3–92.2) |  | 8.3% (2.9–17.4) |  | 2.8% (0.5–8.7) |  |
| Sex |  | 0.813 |  | *0.037* |  | 0.190 |  | 0.353 |
| Female (n=120) | 99.1% (93.6–99.8) |  | 91.0% (82.6–95.5) |  | 8.1% (3.5–15.3) |  | 0.9% (0.1–4.6) |  |
| Male (n=83) | 97.8% (85.6–99.7) |  | 79.3% (65.9–87.9) |  | 14.7% (6.8–25.4) |  | 2.4% (0.5–7.7) |  |
| Dominant site of lesion |  | 0.305 |  | 0.054 |  | *0.011* |  | 0.989 |
| Proximal upper-third/multiple (n=67) | 100% |  | 76.8% (60.4–87.0) |  | 21.7% (10.3–35.8) |  | 1.5% (0.1–7.2) |  |
| Distal two-thirds (n=136) | 97.7% (90.8–99.5) |  | 90.7% (83.1–95.0) |  | 5.5% (2.2–10.9) |  | 1.6% (0.3–5.1) |  |
| Endoscopic type |  | 0.171 |  | *0.043* |  | 0.331 |  | 0.079 |
| Superficial (n=103) | 100% |  | 92.4% (83.4–96.6) |  | 7.6% (2.9–15.2) |  | 0% |  |
| Others (n=100) | 97.2% (88.7–99.3) |  | 80.4% (69.0–88.0) |  | 13.7% (6.8–22.9) |  | 3.1% (0.8–8.1) |  |
| MALT-IPI risk |  | *0.034* |  | 0.810 |  | 0.551 |  | *0.008* |
| Low risk (n=142) | 100% |  | 86.8% (78.3–92.1) |  | 10.9% (5.9–17.8) |  | 0% |  |
| Intermediate to high risk (n=61) | 95.2% (81.5–98.8) |  | 85.3% (70.5–93.0) |  | 9.8% (2.8–21.9) |  | 5.1% (1.3–12.9) |  |
| Lugano stage |  | *<0.001* |  | *<0.001* |  | 0.329 |  | *<0.001* |
| Stage I to II1 (n=181) | 100% |  | 89.6% (82.8–93.9) |  | 7.9% (4.1-13.3) |  | 0% |  |
| Stage II2, IIE, and IV (n=22) | 88.2% (59.9–97.0) |  | 63.9% (38.2–81.2) |  | 17.2% (3.9-38.6) |  | 14.1% (3.3–32.4) |  |
| HPI infection |  | *0.017* |  | *0.004* |  | *0.027* |  | 0.099 |
| Positive (n=151)* | 100% |  | 92.2% (85.7–95.8) |  | 6.5% (3.0–11.9) |  | 0.7% (0.1–3.4) |  |
| Negative (n=52) | 94.3% (78.4–98.6) |  | 70.2% (51.5–82.8) |  | 22.3% (9.9–37.8) |  | 4.1% (0.7–12.6) |  |

CIR, cumulative incidence of relapse; HPI, *Helicobacter pylori* infection; MALT-IPI, mucosa-associated lymphoid tissue lymphoma-International Prognostic Index; DRM, disease-related mortality; OS, overall survival; PFS, progression-free survival

† Univariate analysis variables were selected based on prior literature on the currently known prognostic factors.

* Among 151 HPI-positive patients, 7 (4.6%) were diagnosed with Lugano stage II2, IIE, and IV and received chemotherapy.

**Online Resource 6. Univariate analysis of survival outcomes in primary gastric MALT lymphoma patients (HPI treatment group, n=144)***

| Variables | PFS | *p-*value | CIR | *p-*value |
| --- | --- | --- | --- | --- |
| Age (years) |  | 0.385 |  | 0.708 |
| <60 (n=99) | 94.2% (86.7–97.6) |  | 5.7% (2.1–12.0) |  |
| ≥60 (n=45) | 88.4% (70.7–95.7) |  | 9.3% (2.1–23.0) |  |
| Sex |  | 0.082 |  | 0.179 |
| Female (n=87) | 95.2% (85.1–98.5) |  | 4.8% (1.2–12.7) |  |
| Male (n=57) | 88.2% (75.4–94.5) |  | 9.9% (3.6–20.1) |  |
| Dominant site of lesion |  | *0.043* |  | *0.018* |
| Proximal upper-third/multiple (n=39) | 84.0% (64.6–93.3) |  | 16.0% (5.3–31.8) |  |
| Distal two-thirds (n=105) | 95.6% (88.7–98.3) |  | 3.4% (0.9–8.8) |  |
| Endoscopic type |  | 0.976 |  | 0.672 |
| Superficial (n=78) | 91.9% (81.0–96.7) |  | 8.0% (2.8–16.9) |  |
| Others (n=66) | 93.1% (82.6–97.4) |  | 5.3% (1.4–13.5) |  |
| MALT-IPI risk |  | 0.124 |  | 0.112 |
| Low risk (n=115) | 90.6% (82.5–95.1) |  | 8.4% (3.8–15.3) |  |
| Intermediate to high risk (n=29) | 100% |  | 0% |  |
| Invasion depth by EUS |  | 0.239 |  | 0.233 |
| Mucosa (n=39) | 100% |  | 0% |  |
| Submucosa or beyond (n=27) | 96.1% (75.7–99.5) |  | 3.8% (0.3–16.8) |  |

CIR, cumulative incidence of relapse; HPI, *Helicobacter pylori* infection; MALT-IPI, mucosa-associated lymphoid tissue lymphoma-International Prognostic Index; PFS, progression-free survival

*Among 151 HPI-positive patients, 7 (4.6%) were diagnosed with advanced stage III-IV and received chemotherapy. Therefore, 144 HPI patients who underwent treatment HPI were included in the analysis, and no death events occurred in this group.
